# Role of Phytochemicals in Cancer Prevention

**DOI:** 10.3390/ijms20204981

**Published:** 2019-10-09

**Authors:** Alok Ranjan, Sharavan Ramachandran, Nehal Gupta, Itishree Kaushik, Stephen Wright, Suyash Srivastava, Hiranmoy Das, Sangeeta Srivastava, Sahdeo Prasad, Sanjay K. Srivastava

**Affiliations:** 1 Department of Biomedical Sciences, Texas Tech University Health Sciences Center, Amarillo, TX 79106, USA; alok.ranjan@nih.gov (A.R.); sharvan.ramachandran@ttuhsc.edu (S.R.); nehal.gupta@ttuhsc.edu (N.G.); i.kaushik@ttuhsc.edu (I.K.); stephen.wright@ttuhsc.edu (S.W.); suyash.srivastava17@gmail.com (S.S.); hiranmoy.das@ttuhsc.edu (H.D.); 2 Department of Immunotherapeutics and Biotechnology, and Center for Tumor Immunology and Targeted Cancer Therapy, Texas Tech University Health Sciences Center, Abilene, TX 79601, USA; sahdeo.prasad@ttuhsc.edu; 3 Department of Internal Medicine, Texas Tech University Health Sciences Center, Amarillo, TX 79106, USA; 4 Department of Chemistry, Lucknow University, Mahatma Gandhi Road, Lucknow, UP 226007, India; sangeetas.lu@gmail.com

**Keywords:** chemoprevention, capsaicin, cucurbitacin B, benzyl isothiocyanate, phenethyl isothiocyanate, piperlongumine, isoflavones, catechins, lycopene

## Abstract

The use of synthetic, natural, or biological agents to minimize the occurrence of cancer in healthy individuals is defined as cancer chemoprevention. Chemopreventive agents inhibit the development of cancer either by impeding DNA damage, which leads to malignancy or by reversing or blocking the division of premalignant cells with DNA damage. The benefit of this approach has been demonstrated in clinical trials of breast, prostate, and colon cancer. The continuous increase in cancer cases, failure of conventional chemotherapies to control cancer, and excessive toxicity of chemotherapies clearly demand an alternative approach. The first trial to show benefit of chemoprevention was undertaken in breast cancer patients with the use of tamoxifen, which demonstrated a significant decrease in invasive breast cancer. The success of using chemopreventive agents for protecting the high risk populations from cancer indicates that the strategy is rational and promising. Dietary components such as capsaicin, cucurbitacin B, isoflavones, catechins, lycopenes, benzyl isothiocyanate, phenethyl isothiocyanate, and piperlongumine have demonstrated inhibitory effects on cancer cells indicating that they may serve as chemopreventive agents. In this review, we have addressed the mechanism of chemopreventive and anticancer effects of several natural agents.

## 1. Introduction

Cancer is a disease, which involves abnormal growth of cells with the potential to invade and metastasize to other parts of the body. Among several factors that are involved in cancer initiation include changes in the genes that regulate normal functions of the body. Given the steady increase in cancer incidence worldwide, together with escalating problems with drug resistance, there is increasing interest in various strategies for cancer prevention.

Chemoprevention is the use of natural, synthetic or biological agents to prevent, suppress or to reverse the initial phase of carcinogenesis or to prevent the invading potential of premalignant cells [1]. The interest in the area of chemoprevention has largely increased with growing understanding of the biology of cancer, identification of molecular targets, and success in breast, prostate, and colon cancer prevention [2]. At the molecular level, cancer chemoprevention has been distinguished by alteration of multiple pathways, which play a critical role in the three basic steps of carcinogenesis, that is, initiation, promotion, and progression [3]. Recently, FDA has approved ten new agents for treating precancerous lesions and for reducing the risk of cancer [4].

Clinically, chemoprevention has been categorized into primary, secondary, and tertiary. Primary chemoprevention is suitable for the general population with no cancer, as well as populations at high risk of developing cancer in their lifetime. Secondary chemoprevention is intended for patients with pre-malignant lesions, which may progress to invasive cancer. Generally, primary and secondary chemoprevention has been categorized under primary chemoprevention. Examples of primary chemopreventive agents are dietary phytochemical and non-steroidal anti-inflammatory drugs (NSAID). On the other hand, tertiary chemoprevention is to prevent the recurrence of cancer [5]. For instance, the administration of tamoxifen is an example of tertiary chemoprevention in breast cancer [6]. 

## 2. Capsaicin

Capsaicin (trans-8-methyl-*N*-vanilly l-6-nonenamide) is a pungent alkaloid and active component of chili pepper belonging to the plant genus called *Capsicum* [7,8]. The heat associated with chili pepper is measured in Scoville Heat Units (SHU), which is the factor by which a chili extract is diluted to reduce its heat. The concentration of capsaicin is proportional to the SHU in any given hot chili pepper. The concentration of capsaicin varies from 0.1–1.0% in different peppers.

Capsaicin has been reported as a chemopreventive, tumor suppressing, radiosensitizing, and anticancer agent in various cancer models [9,10,11]. Topical application of capsaicin is used to reduce pain or may represent an effective treatment to alleviate the symptoms of osteoarthritis when oral non-steroidal anti-inflammatory drugs are not used due to side effects [12]. Capsaicin binds to a subfamily of receptor called transient receptor potential cation channel subfamily V member 1 (TRPV1). TRPV1 receptor is also known as capsaicin receptor [13]. In general, anti-cancer activity of capsaicin is not mediated by binding with TRPV1. However, a few studies have demonstrated an increase in intracellular calcium leading to apoptosis upon binding with TRPV1 [13]. Capsaicin treatment blocks the activation of activator protein 1 (AP-1), nuclear factor kappa B (NF-κB), and signal transducer and activator of transcription 3 (STAT3) signaling pathways that are activated and responsible for tumor growth [11]. It has also been shown that capsaicin generates reactive oxygen species (ROS), depolarizes mitochondria or may cause cell cycle arrest leading to apoptosis [11]. Capsaicin reduces bladder cancer cell migration by direct binding with sirtuin 1 (SIRT1) followed by down-regulation of SIRT1 deacetylase [14]. We have demonstrated that capsaicin-induced apoptosis in pancreatic cancer cells was associated with inhibition of β-catenin signaling. Oral administration of 5 mg/kg capsaicin significantly suppressed the growth of implanted pancreatic tumors in mice. After oral administration, within an hour, maximum concentration of capsaicin is achieved in blood and maximum distribution in several organs such as kidneys, lungs, and intestine [15]. 

Capsaicin inhibits the activity of carcinogens, through numerous pathways, and induces apoptosis in several cancer cell lines in vitro and in rodents [7,16,17], and thus may be considered for cancer therapy. The anti-cancer mechanisms of capsaicin are listed in Table 1. However, there have been reports of tumor formation in animals receiving natural capsaicin [18,19]. Studies suggest that compounds contaminating natural capsaicin from peppers may have been responsible for the tumor formation [16]. The cancer enhancement in studies with tumor promoters and carcinogens may have been secondary to the irritating property of capsaicin and may have induced increase blood flow, which may have in turn increased the absorption of the promoters and carcinogens, and thus increased their levels, leading to tumor formation [16]. Direct application with >98% pure capsaicin showed no tumor formation on the skin and all the mice were normal [20]. Several small epidemiological studies suggest a link between capsaicin consumption and stomach or gall bladder cancer, but contamination of capsaicin-containing foods with known carcinogens renders their interpretation problematic [16]. The postulated ability of capsaicin metabolites to damage DNA and promote carcinogenesis remains unsupported [16]. Thus, pure capsaicin appears to be safe and efficacious in animal models, and thus can be evaluated in humans for safety and efficacy against cancer.

In 2014, a phase 2 clinical trial study (NCT02037464) associated with the chemopreventive effect of capsaicin was started. However, the outcome and results of this trial have not been published yet (https://www.clinicaltrials.gov/ct2/show/NCT02037464). The purpose of this study was to evaluate the chemopreventive properties of capsaicin in prostate cancer patients who are enrolled in an active surveillance program or patients scheduled to undergo radical prostatectomy. 

## 3. Catechins

Catechins are natural polyphenols and dietary phytochemicals present in green tea and other beverages [21,22]. Lower incidence of cancer associated with dietary consumption of polyphenols present in plants has been reported [23]. Catechin (C), epicatechin (EC), epigallocatechin (EGC) and epigallocatechin-3-gallate (EGCG) are the major components of green tea [24]. Their concentrations in green tea infusion vary from 9.03–471 mg/L [25]. Catechin is an antioxidant and prevents cardiovascular disease [26,27]. Additionally, catechins have been shown to provide protection against oxidative stress induced by tertbutylhydroperoxide [28,29]. Epigallocatechin gallate (EGCG) is one of the most abundant catechins present in green tea [24]. Furthermore, EGCG has been shown to sensitize cancerous cells to apoptosis induced by anti-cancer drugs and to protect non-cancerous cells from harmful effects of ultraviolet radiation exposure [30]. The anti-cancer effects of catechins are listed in Table 1. 

Dextran-Catechin, a conjugated form of catechin was demonstrated to have better serum stability and was more active against neuroblastoma than unconjugated catechin [46]. Mechanistically, dextran-catechin was observed to induce oxidative stress by decreasing the intracellular glutathione level and by disrupting copper homeostasis [46]. Moreover, catechin extract and nanoemulsion of catechin have been shown to inhibit prostate cancer cells by arresting the cell cycle in *S*-phase, with the half maximal inhibitory concentration being 15.4 μg/mL and 8.5 μg/mL respectively [27]. Additionally, catechins, particularly EGCG, inhibit the proliferation of breast cancer cells by generating reactive oxygen species [29]. EGCG has been demonstrated to have maximum relative efficiency of cellular DNA breakage whereas catechin was reported to possess minimum efficiency [47]. In another study, ribosomal protein S6 kinase (RSK)-2 has been established as a novel molecular target of EGCG using computational docking screening methods [48]. Other studies have suggested that the combination of EGCG and green tea extracts inhibit tumor growth in a xenograft mouse model of several human cancer cell lines. Also, studies have revealed that green tea has chemopreventive properties [49]. In a 10 year prospective cohort study, Drs. Nakachi and Imai showed that drinking 10 cups (120 mL/cup) of green tea everyday delays cancer onset by 7.3 years and 3.2 years in females and males, respectively [50]. Overexpression of ErbB in both normal and mutated forms has been established to play role in cancer metastasis [51]. The study demonstrated that EGCG acts directly or on downstream of ErbB signaling such as mitogen-activated protein kinase (MAPK), STAT and phosphoinositide 3-kinases (PI3K)/AKT/mammalian target of rapamycin (mTOR) pathways [51]. Side effects and acquired resistance associated with conventional platinum based chemotherapy for ovarian cancer is a major drawback [52]. Interestingly, theaflavin-3,3′-digallate (TF3), a monomer present in black tea was demonstrated to induce potent inhibitory effect on cisplatin resistant ovarian cancer cells. Additionally, G2 arrest was shown to be involved in TF3 induced apoptosis in resistant ovarian cancer [31]. Upregulation of p53 via Akt/mouse double minute 2 homolog (MDM2) pathway might be involved in TF3-induced G2 arrest and apoptosis [31].

EGCG was reported to enhance the anti-cancer activity of several anti-cancer drugs such as retinoids [53]. AM80 is a synthetic retinoid that is a clinically used drug for relapsed and intractable acute promyelocytic leukemia patients [53]. A recent study demonstrated that the combination of EGCG and AM80 synergistically induced apoptosis as well as upregulated expression of DNA damage inducible genes such as (GADD153), death receptor 5 (DR5) and p21^waf1^ in lung cancer. Furthermore, downregulation of histone deacetylase 4, -5, and -6 was observed as a mechanism for synergistic induction of apoptosis in lung cancer by EGCG and AM80 [53]. 

Since catechins prevent or slow the growth of prostate cancer, a clinical trial was conducted using green tea catechins for treating patients with prostate cancer undergoing surgery to remove the prostate (NCT00459407). Although the trial started in 2007, results have not been published yet. The primary objective of the study was to estimate the bioavailability of green tea extract in the prostate of patients after the treatment of green tea extract. Furthermore, one of the several secondary objectives was to determine the effect of green tea extract on matrix metalloprotein (MMP)-2 and MMP-9 in prostate cancer patients. 

## 4. Lycopene

Lycopene is a member of the carotenoid family, which is mainly found in tomatoes and other food products such as watermelons, papaya, pink grapefruit, pink guava and red carrot [54,55]. It is a naturally occurring pigment that contributes to the red color in these food products. Lycopene is a potent dietary antioxidant and because of its antioxidant effect, it is known to have a protective effect on several diseases such as cardiovascular diseases, neurodegenerative diseases, hypertension, osteoporosis, diabetes, and cancer [56,57]. The anti-cancer effects of lycopene against a variety of malignancies have been previously discussed by Farzaei et al. [58]. There are about 250 articles available so far on the anti-cancer effects of lycopene. Several anti-cancer mechanisms of lycopene are listed in Table 1. A recent study has been conducted to access the effect of dietary lycopene on prostate cancer. In this study Zu. et al. demonstrated that higher intake of lycopene was associated with lower incidence of prostate cancer. In addition, they found that expression of tumor tissue biomarkers related to angiogenesis, apoptosis, cell proliferation, and differentiation were less in patient samples with higher lycopene intake indicating that lycopene suppresses tumor development by inhibiting tumor neo-angiogenesis [59]. It has been reported that lycopene tends to preferentially accumulate in prostate tissue as compared with other tissues, which might be responsible for its anti-prostate cancer activity [54]. Several other studies have shown that lycopene causes cell cycle arrest and apoptosis in prostate cancer cells [60,61]. Moreover, lycopene inhibits the growth of prostate and breast cancer cells by inhibiting NF-κB signaling [62]. A study by Chen et al. showed the anti-angiogenic activity of lycopene in both in vitro and in vivo models, proposing that the mechanism of action may involve modulation of PI3K-Akt and ERK/p38 signaling pathways [32]. 

Several studies have shown that lycopene in combination with melatonin shows strong chemopreventive activity via antioxidant and anti-inflammatory activities [63,64,65]. Lycopene also enhances the effect of quinacrine on breast cancer cells by inhibiting Wnt-TCF signaling [33]. Oral administration of 16 mg/kg lycopene for 7 weeks significantly inhibited prostate tumor growth by 67% when compared to control in athymic nude mice. The study also showed that lycopene reduced the expression of proliferating cell nuclear antigen (PCNA) and VEGF in tumor tissues and plasma respectively [66].

Several clinical trials have been commenced to investigate the chemopreventive and chemotherapeutic effects of lycopene on the progression of prostate cancer. Nonetheless, studies report conflicting beneficial effects of lycopene in reducing prostate enlargement and decreasing serum prostate-specific antigen (PSA) levels whereas others studies have null findings. (NCT00006078, NCT01443026, NCT00068731). In a randomized clinical trial, administration of 15 mg lycopene every day for 6 months in benign prostate hyperplasia patients resulted in reduced disease progression with decreased serum PSA concentrations [67]. 

## 5. Cucurbitacin B

Cucurbitacins are tetracyclic triterpenoids that are found in traditional Chinese medicinal plants belonging to the cucurbitaceae family. Among eight different types of Cucurbitacins, Curcubitacin B (CuB) is the most active component against cancer and showed promise in various cancer models [68].

The effective concentrations of CuB in vitro range from 20 nM–5 µM and in vivo therapeutic doses range from 0.1–2 mg/kg [69]. Various anti-cancer mechanisms of CuB are mentioned in Table 1. Several studies have shown that CuB inhibits STAT3 signaling in various cancer models such as colorectal cancer [34], lung cancer [70], neuroblastoma [35], acute myeloid leukemia [71], pancreatic cancer [72] and breast cancer [36]. Recent studies have established the anti-angiogenic effects of CuB associated with inhibition of VEGF/FAK/MMP-9 signaling in highly metastatic breast cancer cells [73]. In non-small cell lung cancer, the anti-metastatic effect of CuB was achieved by targeting the Wnt/β-catenin signaling axis [74]. A study from our laboratory demonstrated that CuB inhibits breast tumor growth by inhibiting HER2-intergrin signaling. The inhibition of HER2-integrin signaling was associated with down regulation of integrin α6 and integrin β4 that are overexpressed in breast cancer cells [36]. In addition, it has been reported that CuB reduces invasion and migration of hepatoma cells by modulating PI3K/Akt signaling [75]. Furthermore, several studies demonstrated the potentiating effect of CuB with other chemotherapeutic agents. In pancreatic cancer, CuB augmented the anti-proliferative effects of gemcitabine by inhibiting JAK-STAT pathway [76]. CuB was also shown to sensitize cisplatin-resistant ovarian cancer cells to apoptosis when combined with cisplatin, a standard chemotherapeutic agent for ovarian cancer [77]. Another study demonstrated that CuB in combination with docetaxel or gemcitabine synergistically suppressed the growth of breast cancer cells [78]. Interestingly, combination of CuB with curcumin in hepatoma cells reversed multidrug resistance by modulating P-gp [79].

Studies have been conducted to compare the pharmacokinetic profile of CuB with that of CuB loaded solid lipid nanoparticles. The plasma AUC of CuB loaded nanoparticles was 2.47 µg·h/mL, which was almost 2-fold higher than plasma AUC of CuB (1.27 µg·h/mL) after an intravenous dose of 2 mg/kg. It was observed that CuB loaded nanoparticles showed 3.4 fold increased uptake in tumor cells when compared with CuB and exhibited better tumor suppressive effects [80]. CuB was mainly distributed in organs such as spleen and liver. Another study has demonstrated that CuB loaded modified phospholipid complex improved therapeutic efficacy, bioavailability and targeted drug delivery for cholangiocarcinoma [81].

## 6. Benzyl Isothiocyanate (BITC)

Isothiocyanates (ITCs) are natural compounds of high medicinal value that are present in cruciferous vegetables such as broccoli, watercress, Brussels sprouts, cabbage, cauliflower and Japanese radish [82]. They are present as conjugates in the genus *Brassica* of cruciferous vegetables [38]. ITCs are well-known for their chemo-preventive activity and mediate anti-carcinogenic activity by suppressing the activation of carcinogens and increasing their detoxification [82]. The high content of glucosinolates, which store ITCs in cruciferous vegetables confer anti-cancerous effects. ITCs suppresses tumor growth by induction of oxidative stress mediated apoptosis, inducing cell cycle arrest, inhibiting angiogenesis and metastasis [82]. 

Benzyl isothiocyanate (BITC) is one of the major classes of ITCs that exert potential health benefits to humans. It is extensively found in *Alliaria petiolata*, pilu oil, water cress, garden cress and papaya seeds [83]. BITC found in *Salvadora persica* has been shown to exert anti-bacterial activity against Gram-negative bacteria [84]. BITC influences several key signaling pathways which are considered to be the hallmarks of cancer. In addition, BITC sensitize tumors to chemotherapy and has substantial anticancer effects against various human malignancies like leukemia [85], breast cancer [86], prostate cancer [87], lung cancer [88], pancreatic cancer [89] colon cancer [38] and hepatocellular carcinoma [90] as mentioned in Table 1. A published study demonstrated that BITC induces DNA damage in human pancreatic cells. It was also shown that DNA damage causes G_2_/M Cell cycle arrest and apoptosis [37]. Another study established BITC mediated inhibition of the migration and invasion of human colon cancer cells. The anti-invasive effect of BITC was through down-regulation of MMP-2/9 and urokinase-type plasminogen activator (uPA) linked to protein kinase C (PKC) and MAPK signaling pathways [38]. In our previous study, we have shown that BITC induces apoptosis in pancreatic cancer cells but not in normal human pancreatic ductal epithelial cells. The induction of apoptosis by BITC was through inhibition of STAT3 signaling. In the same study, oral administration of 12 µmol BITC significantly suppressed the growth of BxPC3 pancreatic tumor xenograft in athymic nude mice [89]. In another study, we have demonstrated that BITC suppressed pancreatic tumor growth by inhibiting PI3K/AKT/FOXO pathway [39]. We have also demonstrated that BITC suppresses angiogenesis and invasion in pancreatic tumors by inhibiting STAT3 mediated HIF-1α/VEGF/Rho-GTPases [40]. BITC also displayed antitumor effects by potentiating p53 signaling in breast cancer cells. p53 activation was through the activation of p53-LKB1 and p73-LKB1 axes. In the same study, it was also reported that BITC suppressed the mammosphere –forming capability of breast cancer cells [91]. 

Our studies have shown that BITC possesses therapeutic selectivity towards cancer cells and does not affect normal human pancreatic epithelial cells. BITC was detected in pancreatic tumors and plasma, indicating that the therapeutic concentration can be achieved by oral administration [39]. The concentration of BITC achieved in tumor tissue and plasma was 7.5 µmol/g and 6.5 µmol/L respectively after oral administration of 12 µmol BITC in athymic nude mice [39]. In one of our published studies, nano-emulsion BITC was prepared to enhance its dissolution and solubility. The entrapment efficiency of BITC nano-emulsion was observed to be 15–17 mg/mL leading to increased accumulation in the tumor cells [92]. 

## 7. Phenethyl Isothiocyanate

Phenethyl isothiocyanate (PEITC) is another isothiocyanate mainly present in cruciferous plants. PEITC is one of the active ingredients of cruciferous vegetables that have been extensively studied for its anti-cancer effects in glioblastoma, prostate cancer, breast cancer and leukemia [36] and listed in Table 1. Several studies have indicated that consumption of cruciferous vegetables such as broccoli, watercress, and garden cress leads to chemoprevention in various rodent models [93]. A study demonstrated RASSF1A reactivation by PEITC, which is known to have tumor suppressive functions by promoting G2/M cell cycle arrest and apoptosis in prostate cancer cells [42]. Our study established for the first time the anti-metastatic potential of PEITC in a breast cancer model. Our results showed that oral administration of 10 µmol PEITC for 10 days suppressed the metastasis of breast tumor cells to the brain [94]. Another study by us indicated HER2 as a potential target of PEITC in breast carcinoma. PEITC exhibited synergistic effect when combined with doxorubicin and was associated with down regulation of HER2 and STAT3 [41]. PEITC was also shown to induce ROS generation in p53-deficient chronic lymphocytic leukemia cells (CLL) and therefore could be effective for treatment of CLL patients with p53 mutations [95]. Interestingly, the combination of PEITC and paclitaxel synergistically potentiated the anti-proliferative effects of paclitaxel on breast cancer cells.by inducing apoptosis and cell cycle arrest [96]. It has been reported that PEITC in combination with adraimycin or etoposide causes caspase 3 and 8 activation by modulating PKCs and telomerase and thus sensitizes the cervical cancer cells [97]. A recent study showed chemopreventive effects of PEITC and curcumin combination in prostate cancer xenografts [98]. Our lab has shown the immune modulation by PEITC in mice bearing breast tumor xenografts. We observed that PEITC treatment significantly suppressed breast tumor growth by reducing myeloid derived tumor suppressor cells (MDSCs) and T regulatory lymphocytes [99]. 

PEITC is fairly lipophilic in nature with a molecular weight of 163.2 g/mol [100,101]. Pharmacokinetics of PEITC is well established in rodents as well as in humans. Ji et al. [102] performed a detailed pharmacokinetic study of PEITC in Sprague-Dawley rats. At a dose of 10 µmol/kg (1.63 mg/kg), oral bioavailability of PEITC was 115%. The apparent volume of distribution (V_d_) and clearance were 1.94 ± 0.42 L/kg and 0.70 ± 0.17 L/h/kg, respectively at the dose of 2 µmol/kg PEITC. In another clinical study, 100 g of watercress was given to four human volunteers and plasma concentration was determined using one-compartment pharmacokinetic model [103]. The highest plasma concentration (C_max_) attained was 928.5 nM as estimated by LC-MS/MS with Tmax and T_1/2_ around 2.6 h and 4.9 h, respectively.

A phase II clinical trial study (NCT00691132) for chemopreventive effects of PEITC against lung cancer started in 2009 and completed in the year 2013. The primary end point of this study was to determine whether PEITC is effective in preventing lung cancer in cigarette smokers. The metabolic activation of tobacco carcinogen 4-(methylnitrosamino)-1-(3-pyridyl)-1-butanone (NNK) was reduced by 7.7% with PEITC treatment in smokers [104]. Another study started in year 2011 by National Cancer Institute (NCT01265953) to evaluate the chemopreventive effects of PEITC against prostate cancer. From this clinical trial, it was found that isothiocyanate inhibits histone deacetylase (HDAC) activity in human colorectal and prostate cancer cells.

## 8. Isoflavones

Isoflavones are naturally occurring isoflavonoids present in plants belonging to the leguminosae family [105]. Isoflavones are extensively present in soy, lentil, bean, chickpeas and have profound importance as phytoestrogens in mammals. Soy is an abundant source of isoflavones, such as, genistein, glycitein, and daidzein, the concentration of which varies between 560 and 3810 mg per kg of soy [106]. Isoflavones are present in inactive form as glycosides in plants and are activated to bioactive aglycones by hydrolyzation to beta-glucosidases in the intestine. The aglycones are conjugated to liver glucorinides and excreted in urine [107]. Interestingly, the active form of isoflavones has a greater absorption rate than inactive form. 

Isoflavones exert potential health benefits and are widely used in the treatment of hormone dependent conditions like menopause, cardiovascular disease, osteoporosis, and cancer [105]. Isoflavones derived from soy, such as genistein, have been established to have significant anti-cancer effects against leukemia, lymphoma, gastric, breast, prostate and non-small cell lung cancer [44]. Several studies have reported the anti-cancer effects of genistein in various cancer models such as prostate cancer [108], breast cancer [109], lung cancer [110] and head and neck squamous cell carcinoma [111], cervical cancer [112], ovarian cancer [113], renal cancer [114], bladder cancer [115], liver cancer [116] as shown in Table 1. Induction of apoptosis by genistein treatment was shown through inhibition of IGF-1R/p-Akt signaling in breast cancer [43]. Another study demonstrated anti-angiogenic and anti-metastatic effects of genistein by inhibiting c-erbB-2, MMP-2, and MMP-9 in breast carcinoma [44]. Genistein has been reported to induce differentiation in breast cancer stem cells by interaction with ER+ cells. This differentiation effect of genistein is mediated by the PI3K/Akt pathway [95]. Soy isoflavones are capable of sensitizing the cells to radiotherapy, thereby improving the efficacy of current treatment [117]. It has been demonstrated that soy isoflavones overcome radiotherapy resistance by inhibiting the altered activation of APE1/Ref-1, NF-κB, and HIF-1α [118]. Additionally, genistein has also been reported to induce anti-oxidant properties [113,119,120]. Isoflavones such as genistein and daidzein have minimal clinical toxicity [121]. 

A clinical trial using purified isoflavones was started in 2009 (NCT01036321) and completed in 2018. The main focus of this trial was to compare safety, effectiveness, and mechanism of action of purified isoflavones in African American and Caucasian Mento with prostate cancer. Change in percent Ki-67 was evaluated in prostate tumor tissues after 3–6 weeks of intervention with purified isoflavones (40 mg daily) vs. Placebo. On the basis of this clinical trial outcome, isoflavones could be developed as a potential chemotherapeutic and chemopreventive agent. 

## 9. Piperlongumine

Piperlongumine or Piplartine (5,6-dihydro-1-[(2E)-1-oxo-3-(3,4,5-trimethoxyphenyl)-2-propenyl]-2(1H)-pyridinone) is a phytochemical alkaloid extracted from the roots of long pepper Piper longum L., a member of the Piperaceae family. Long peppers have profound medicinal importance in Indian Ayurvedic medicine and Latin American folk medicine [122]. Piperlongumine was used to treat various diseases such as bronchitis, malaria, viral hepatitis, cancer, and melanogenesis [123]. The key therapeutic features of piperlongumine are its anti-inflammatory, anti-nociceptive, anti-bacterial, anti-fungal, anti-diabetic, anti-tumor, and anti-depressant properties [122]. Overall, piperlongumine has significant chemotherapeutic and chemopreventive potential making it an effective treatment option for cancer. 

Piperlongumine has been found to be effective against several cancers such as multiple myeloma [124], melanoma [125], pancreatic cancer [126], colon cancer [127,128] oral squamous cell carcinoma [129], non-small-cell lung cancer [130], gastric cancer [131], biliary cancer [132], and prostate cancer [133]. The mechanism of the anti-cancer effects of piperlongumine is listed in Table 1. Piperlongumine induced ROS generation leads to oxidative stress mediated DNA damage in pancreatic cancer cells [126]. The study reveals that piperlongumine induces autophagy-mediated apoptosis by inhibition of PI3K/Akt/mTOR in lung cancer [45]. It also inhibits inflammation by suppressing inflammatory transcription factors NF-κB [127]. We have demonstrated that piperlongumine inhibits STAT3 and its activation to suppress anoikis resistance resulting in inhibition of metastatic potential of pancreatic cancer and melanoma [125,134]. Furthermore, piperlongumine has been reported to display synergestic effect with paclitaxel or cisplatin in human ovarian cancer cells [135].

The toxicity and pharmacokinetic profile of piperlongumine have been well established. Piperlongumine treated rats and mice with doses varying from 100–3000 mg/kg did not show any signs of toxicity. After oral administration of 5 mg/kg and 10 mg/kg piperlongumine, the t_1/2_ was found to be 1.42 h and 0.84 h and Cmax was 884.31 µg/L and 201.42 µg/L respectively [122]. Our lab has shown that nano-emulsion of piperlongumine enhanced its bioavailability and efficacy [136]. In conclusion, piperlongumine has been established to be an effective agent for cancer treatment.

## 10. Conclusions

**Figure 1:**
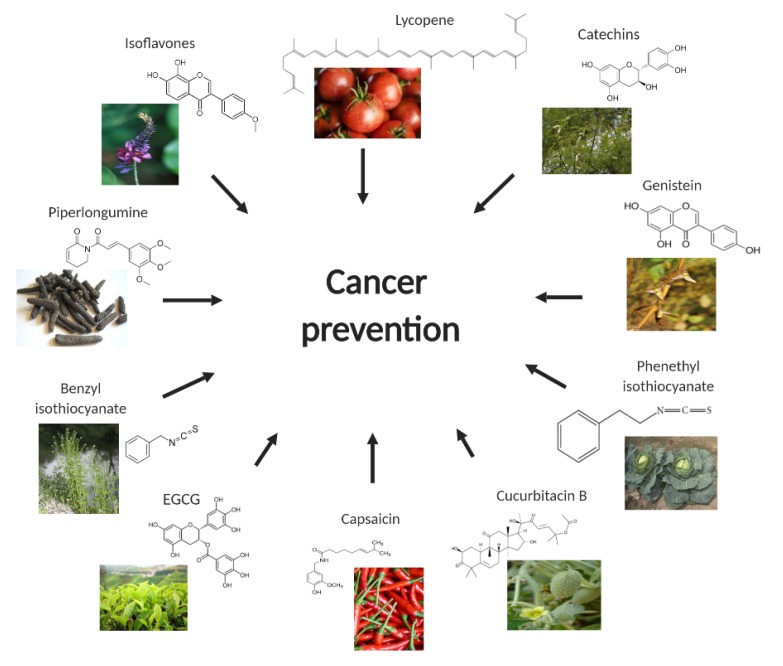
Phytochemicals in cancer chemoprevention.

Chemoprevention is a relatively safe and cost effective approach because cancer can be prevented by changing dietary habits [137]. This approach has gained momentum after the approval of tamoxifen and raloxifen by US Food and Drug Administration for breast cancer risk reduction [138]. Various epidemiological and preclinical studies have convincingly argued the role of several dietary agents to be involved in preventing occurrence of cancer as well as its treatment. Several clinical trials associated with chemopreventive properties of above discussed natural compounds are ongoing. Drug associated toxicity is a significant barrier for currently available chemotherapeutic drugs. However, use of natural compounds for cancer prevention may mitigate associated toxicity. However, bioavailability is the biggest problem with most of the naturally occurring chemopreventive agents. Overall, this review summarizes natural compounds targeting different signaling pathways involved in cancer progression, suggesting their potential to be successful anti-cancer agents (Figure 1).

## Figures and Tables

**Table 1:** Summary of the mechanisms of action of various phytochemicals in various cancer models.

Compound	Source	Cancer	Proposed Anticancer Mechanism	Reference
**Capsaicin** 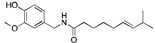	Chilli pepper (Capsicum)	Pancreatic cancer	Blocks AP1, NF-κB and STAT3 signaling, cell cycle arrest, inhibition of β-catenin signaling	[7,11]
**Catechins** 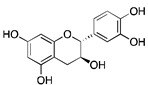	Green tea and other beverages	Neuroblastoma, Breast cancer, Prostate cancer	Cell cycle at G2 phase, protection against oxidative stress, Affecting STAT3-NFκB and PI3K/AKT/mTOR pathways	[27,31]
**Lycopene** 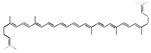	Tomatoes, papaya, pink grapefruit, pink guava, red carrot	Prostate cancer, Breast cancer, cervical cancer	Dietary Antioxidant, Affecting NF-κB signal transduction, Antiangiogenic effect, Inhibition of Wnt-TCF signaling	[32,33]
**CucurbitacinB** 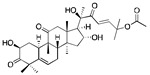	Medicinal plants (Cucurbitaceae family)	Colorectal cancer, Lung cancer, Neuroblastoma, Breast cancer, Pancreatic cancer	Inhibitors of JAK-STAT3, HER2-integrin, and MAPK signaling pathways	[34,35,36]
**Benzyl isothiocyanate (BITC)** 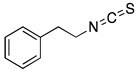	*Alliaria petiolata*, pilu oil, papaya seeds	Leukemia, Breast cancer, Prostate cancer, Lung cancer, Pancreatic cancer, Colon cancer, Hepatocellular carcinoma	G_2_/M Cell cycle arrest and apoptosis, down-regulation of MMP-2/9 through PKC and MAPK signaling pathway, inhibition of PI3K/AKT/FOXO pathway, STAT3 mediated HIF-1α/VEGF/Rho-GTPases inhibition	[37,38,39,40]
**PEITC** 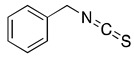	Cruciferous vegetables	Glioblastoma, Prostate cancer, Breast cancer, Cervical cancer, and Leukemia	ROS Activation, G2/M cell cycle arrest, and apoptosis, down regulation of HER2 and STAT3 signaling,	[41,42]
**Isoflavone** 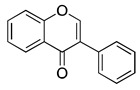	Soy, lentils, beans, and chickpeas	Leukemia, Lymphoma, Gastric, Breast, Prostate, Head and Neck carcinoma, and Non-Small Cell Lung Cancer	Inhibition of c-erB-2, MMP-2, and MMP-9 signaling pathways, Affecting IGF-1R/p-Akt signaling transduction	[43,44]
**Piperlongumine** 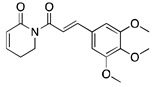	Roots of long pepper	Multiple myeloma, melanoma, Pancreatic cancer, colon cancer, Oral squamous cell carcinoma, Breast cancer, and Prostate cancer	Autophagy-mediated apoptosis by inhibition of PIK3/Akt/mTOR	[45]
